# Study of status of safe injection practice and knowledge regarding injection safety among primary health care workers in Baglung district, western Nepal

**DOI:** 10.1186/1472-698X-13-3

**Published:** 2013-01-03

**Authors:** Sudesh Gyawali, Devendra S Rathore, Bhuvan KC, P Ravi Shankar

**Affiliations:** 1 PhD research Scholar, School of Pharmacy, Suresh Gyan Vihar University, Jaipur, India & Assistant Professor, Department of Pharmacology, Manipal College of Medical Sciences (MCOMS), Pokhara, Nepal; 2 Department of Pharmacy, Rajasthan Pharmacy College, Jaipur, India; 3 Department of Pharmacy, Novel Academy, Pokhara Sub-metropolitan city, Kaski, Nepal; 4 Department of Pharmacology, KIST Medical College, Lalitpur, Nepal

**Keywords:** Baglung, Health care workers, Needle stick injury, Nepal, Safety, Safe injection practice, Sharp waste

## Abstract

**Background:**

Unsafe injection practices and injection overuse are widespread in developing countries harming the patient and inviting risks to the health care workers. In Nepal, there is a dearth of documented information about injection practices so the present study was carried out: a) to determine whether the selected government health facilities satisfy the conditions for safe injections in terms of staff training, availability of sterile injectable equipment and their proper disposal after use and b) to assess knowledge and attitudes of healthcare workers in these health care facilities with regard to injection safety.

**Methodology:**

A descriptive cross-sectional mixed type (qualitative and quantitative) survey was carried out from 18^th^ May to 16^th^ June 2012. In-depth interviews with the in-charges were conducted using a semi-structured questionnaire. Observation of the health facilities using a structured observation tool was done. The data were analysed manually by summarizing, tabulating and presenting in various formats.

**Results:**

The in-charges (eight males, two females) who participated in the study ranged in age from 30 to 50 years with a mean age of 37.8 years. Severe infection followed by pain was the most important cause for injection use with injection Gentamicin being most commonly prescribed. New single use (disposable) injections and auto-disable syringes were used to inject curative drugs and vaccines respectively. Sufficient safety boxes were also supplied to dispose the used syringe. All health care workers had received full course of Hepatitis B vaccine and were knowledgeable about at least one pathogen transmitted through unsafe injection practices. Injection safety management policy and waste disposal guideline was not available for viewing in any of the facilities. The office staff who disposed the bio-medical wastes did so without taking any safety measures. Moreover, none of these staff had received any formal training in waste management.

**Conclusions:**

Certain safe injection practices were noticed in the studied health care facilities but there remain a number of grey areas where unsafe practices still persists placing patient and health workers at risk of associated hazards. Training concentrating on injection safety, guidelines to dispose biomedical waste and monitoring of the activity is needed.

## Background

In developing countries, the use of injections for management of serious and even minor medical problems is common and often unnecessary and they are used unsafely [1,2]. Studies have shown that the degree of unsafe use of injections was highest (75%) in the South East Asia Region including Nepal [1]. ″The global burden of disease, due to unsafe injection use, estimated by the World Health Organization (WHO) by probability model for the year 2008 was 340,000 Human Immunodeficiency Virus (HIV) infections, 15 million Hepatitis B Virus (HBV) infections, 1 million Hepatitis C Virus (HCV) infections, 3 million bacterial infections and 850,000 injection site infections. This accounted for 14% of HIV, 25% HBV, 8% HCV and 5% of bacterial infections worldwide and for 28 million preventable disability adjusted life years″ [3]. The spread of blood borne viral diseases through sexual and vertical means is decreasing, while their transmission by unsafe injectable use is assumed to be increasing [4]. This excess and unsafe use of injections has financial impact as well. Injections are not affordable by many poor families of developing countries as many of them are even unable to satisfy their basic needs- food, shelter and housing.

Unsafe injection practices not only harm the patient but also carry risks to the health care workers (HCWs). Needle stick injury (NSI) is commonly encountered by the provider. About thirty different infectious diseases can be transmitted by NSI [5] among which the chances of acquiring hepatitis B infection is much higher than other infections. Unfortunately, in developing countries, less number of HCWs are vaccinated against Hepatitis B [6] and they work in adverse conditions where occupational hazards are very high compared to western countries [7].

**Figure 1:**
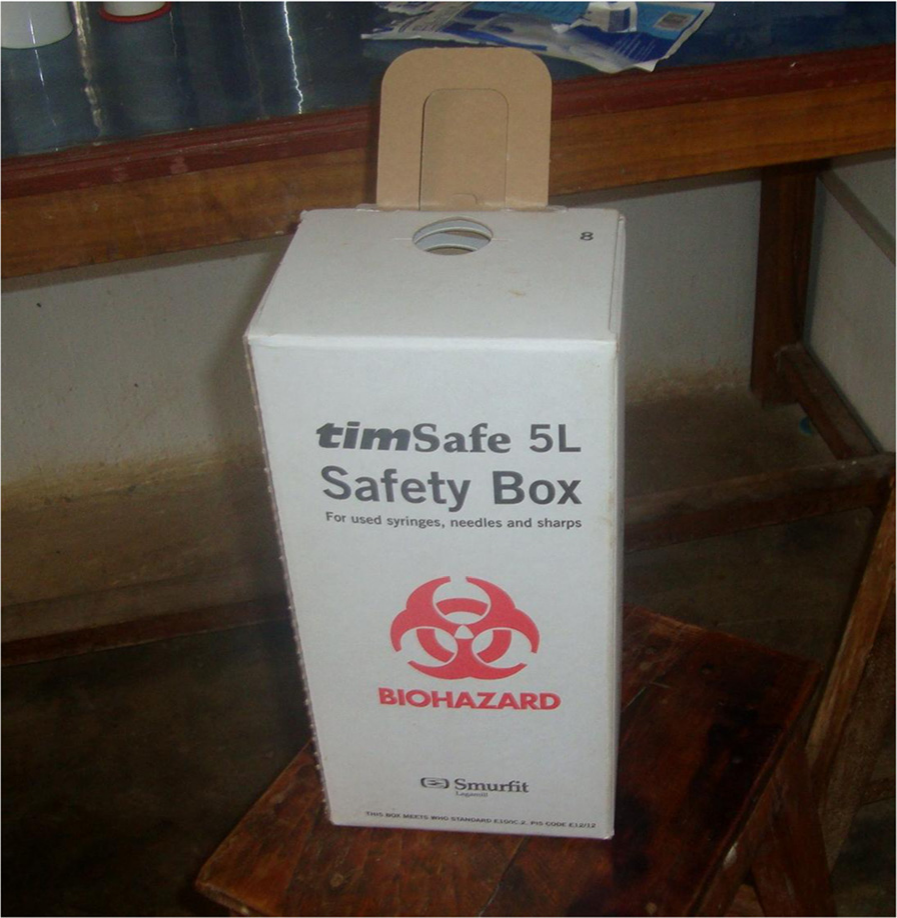
Safety box used for disposal of syringes.

A safe injection is one that, ″does not harm the recipient (patient), does not expose the provider (HCWs) to any avoidable risk and does not result in waste that is dangerous for the community″ [8,9]. Hence, safe injection practice involves administration of rational injection by a qualified and well trained person using a sterile device (syringe, needle etc), adopting sterile technique, and discarding the used devices in a puncture-proof specially designed container for appropriate disposal. Any breach in the process makes the injections extremely unsafe and hazardous to HCWs (provider) as well [9]. In order to achieve safe injection practice, Government of Nepal has been trying to deliver health care services by mobilizing qualified health personnel, ensuring availability of sufficient quantity of sterile devices including auto-disable syringe for immunization and safety boxes (puncture-proof specially designed container for appropriate disposal of syringes) Figure 1. Auto-disable syringe are manufactured in such a way that they cannot be reused. They include a mechanism to immobilize the plunger or block the needle or cause the syringe to leak when a second injection is attempted. The needle is also fixed permanently in the syringe to prevent the reuse of the needle as well [10].

In the present Nepalese health care system, a sub health post (SHP) is the first institutional contact point for basic health services. Above this is the health post (HP). In both health facilities, there may be a birthing centre attached. In these facilities, a health assistant (HA) or an auxiliary health worker (AHW) provides basic health service assisted by auxiliary nursing midwife (ANM) and an office helper. The HAs and the AHWs undergo basic medical training for 36 months and 18 months respectively after 10 years of schooling. Likewise, the ANMs obtain basic nursing training of 18 months after 10 years of schooling and generally they assist in delivery of child. The primary health centre (PHC) is the highest contact point for patients in rural and remote locations and in the healthcare hierarchy it lies above HP and SHP. The PHC is run by a medical doctor who is assisted by other paramedical staff namely HA, AHW, ANM etc.

Most interventions for promotion of injection safety include three parts a) make patients more informed consumers of health care services so that they demand safe injections b) promote occupation safety of HCWs and c) safe disposal of injection equipment (environmental aspect). Even though, all three parts are vital for promotion of safe injection practice, safety of HCWs is of prime importance [7]. The prevailing health care systems of developing countries like Nepal are more health worker centred. It indicates that the HCWs have much greater control and influential role to play [11]. The patients, especially from rural area, tend to take a passive role when interacting with HCWs, have faith in them and accept their professional judgement [7,12]. The informed primary HCWs may have confidence and encouragement for safe injection practice which may change their attitude towards HIV/AIDS patients. If they feel protected from the risk of occupational infections, they may discriminate less against patients with HIV/AIDS [11]. These informed HCWs are in a better position to deliver community education regarding safe injection practice [7].

In the context of Nepal, there is a dearth of documented information and studies on injection safety. Although a few studies on injection safety have been conducted by non-governmental organizations (NGOs), they are accessible in the abstract form only [13]. Hence, this study was carried out among HCWs working in government primary health facilities of Baglung district of western Nepal. This study was conducted with the following objectives:

1. To determine whether the selected governmental health facilities of Baglung district satisfy the conditions for safe injections in terms of staff training, availability of sterile injectable equipment and their proper disposal after use and

2. To assess knowledge and attitudes of healthcare workers in these health facilities with regard to injection safety.

## Methods

### Study area

The research was carried out among HCWs from different village development committees (VDCs) of Baglung district. Baglung district is situated in the hilly region of western Nepal with a total population of approximately 300 thousand. The literacy rate is 70 per cent and the population is of mixed ethnicity. The Baglung district health office regulates 1 district health office, 3 PHCs, 9 HPs and 49 SHPs. Beside these, the Dhaulagiri regional hospital is also situated in Baglung district [14].

### Study design and procedure

A descriptive cross-sectional mixed type (qualitative and quantitative) survey was carried out from 18^th^ May to 16^th^ June 2012. As the facilities and healthcare worker involved in the primary health centres are almost similar (homogeneous) in nature and the study was a preliminary one, ten centres were selected randomly out of 58 primary health care facilities. Names of all primary health facilities were written on cards and were shuffled. Then one was drawn out and the name was noted. After replacing the card drawn, it was reshuffled and the second card was drawn. The process was repeated till ten names were chosen. The card that was drawn for second time was rejected. In-charges of health centres present at the time of visit were requested to participate in the study. They were explained that the topic of research interest was infection control practice, rather than injection practice, so that they were not unduly self-conscious during injection related procedures. Written informed consent was obtained from the interested in-charges of respective centres. They were given the options to accept or refuse to participate. The gender, experience and qualification of the participants were noted. Ethical approval was obtained from Nepal Health Research Council.

### Data collection tools and techniques

In-depth interviews with the in-charges were conducted using a semi structured questionnaire. Observation of the health facilities using a structured observation tool was done.

The data collection tools developed by WHO [15] were modified to a small extent by the authors through consensus. The questionnaires were translated into Nepali and the interview was conducted in Nepali language. The data collection was done by two authors (SG and BKC). The answers of the respondent with reasoning were noted immediately in the answer sheets. The data were analysed manually by summarizing, tabulating/labelling and were presented in various formats.

## Results and discussion

Ten selected primary health facilities were visited and observed. All the in-charges (8 males and 2 females) participated in the study. They ranged in age from 30 to 50 years with a mean age of 37.8 (SD = 5.61) years. The mean work experience was 14 (SD = 5.25) years. Nine of them were AHW and one was ANM. Average number of patients visiting the facility were 20.7 (SD = 3.94) per day and out of these, less than one patient received at least one injection. The figure does not include the vaccinations given during national vaccination campaigns. The HCWs were of the opinion that they do not prescribe more injections because the number of injections (type) supplied by government is less and oral formulations are safer. Most of them (90%) feel that oral formulations are preferred by the patient as well.

The HCWs of primary health facilities generally diagnose and manage minor ailments using few essential drugs supplied by the government. These drugs are distributed free of cost and contain lesser number of injectable. This might be one of the important reasons for lesser number of injections used. Furthermore, these HCWs do not get any economic incentive to prescribe injections because they earn a fixed salary which is independent of their prescribing behaviour. This finding corresponds to those noted in other studies [1,12]. It was observed that a few village medical shops located close to the health centres were run by the same HCWs working in the government health centres and were used to carry out their private practice. The possibilities of excessive use of injection by them in their private clinics for economic benefit [16] could not be excluded.

When the respondents were asked for names of three most common diseases/conditions for which injection is used, 9 (90%) respondents said severe infections (including pneumonia, enteric fever) is one of the most important diseases. Similarly 4 (40%) said pain (including back pain, abdominal pain); 3 (30%) said for contraception and 3 (30%) said for delivery. Gentamicin, diclofenac, depoprovera, ceftriaxone and oxytocin were the five most commonly used injections in the surveyed government primary health care facilities. The antibiotics (Gentamicin and Ceftriaxone) and pain killer (diclofenac) were the most commonly prescribed injections in our study which was comparable to the study carried out in Bangladesh where injectable antibiotics were most commonly (78.3%) prescribed followed by intravenous fluids and pain killers (29.4%) [17].

Among those drugs, gentamicin, depoprovera and oxytocin are supplied free from the district health office to the health care facilities and are included in the government essential drug list. Diclofenac is supplied free of cost only for PHC not for HP and SHP whereas ceftriaxone is not supplied at all but is in the complementary section of the essential drug list of Nepal [18].

### Risk to patients

According to the respondents (in their opinion), they administer more injections as vaccines than as treatment for illnesses. New single use (disposable) injection equipment was used in all the health facilities. For vaccination auto-disable syringes were used. Continuous availability of sufficient quantity of injection equipment is crucial to ensure safe injection practice [19]. Shortage of single use injectable device leads to reuse of the device [9,19]. It was encouraging to find continuous availability of sufficient quantities of injection equipment and no evidence of its reuse. Vaccines were supplied to the health centres with corresponding quantities of injection equipment. For therapeutic injections, the injection equipment did not match the quantity of injectable drugs but was sufficient for single use. As the health centres were managed by the government, all the injections (vaccine and therapeutics) were provided free of cost.

In Nepal, except one syringe manufacturing company, none of the pharmaceutical companies manufacture injection equipment. Hence, this equipment is imported mostly from India and China. Department of Drug Administration is the only regulatory body which ensures the quality of drugs manufactured and/or imported in Nepal. However, the concerned government body is not involved in monitoring the quality of surgical equipment, including injecting equipment. In fact there is no legally authorised body in Nepal to ensure the quality of these equipment. Furthermore, a few studies indicate resale (reuse) of single use disposable injectable equipment (including syringes) [2,9,12,20]. In the absence of a defined monitoring body and the reported incidence of malpractice and resale of injectable equipment, the quality of the syringes available in Nepal may be doubtful.

### Risk to health care workers (HCWs)

Proper disposal of injection equipment is required not only to check the reuse & resale of disposable syringes, but also to minimize the chances of avoidable risks to HCWs [21]. Needle burners and cutters are small device that burn the tip of the needle and then cut the tip of the syringe making both unusable. These devices were not available in any of the health centres so were not used by the HCWs. Though these devices seem an appealing solution to check the reuse of syringes and protect HCWs from NSI, practically there are many challenges associated with their use namely cost, quality and maintenance of device. Furthermore, ensuring uninterrupted electric power supply to operate the machine is a major challenge in Nepal. Disposal of syringe along with needle after use in a puncture proof closed container (safety box) Figure 1 and burning it after two third of the box is full is a good alternative to the use of such device.

**Figure 2:**
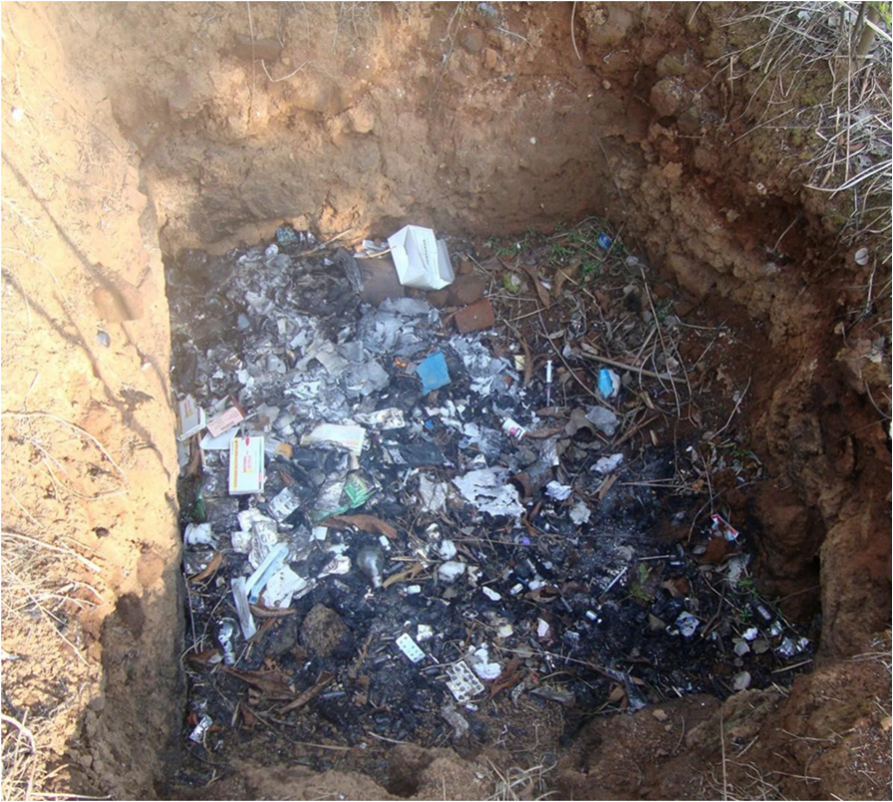
Syringe found in disposal pit (other than safety box).

Use of safety box for disposal of used syringe is important for the safety of HCWs. In our study, it was witnessed that safety boxes were present in stock in all health facilities. All the in-charges reported that the boxes were supplied by the government in sufficient quantity and they dispose the syringe in a safety box immediately after use without recapping. In contrast, in most of the facilities the used syringes (with recapped needles) and other sharps devices were present in open boxes or in other unsafe containers or even in pits Figure 2 especially assigned for burning (disposal) of biomedical wastes discarded from the facility. The presence of sharps, needles and syringes in open boxes or in other unsafe containers in the facilities indicate lack of compliance.

Needle stick injuries (NSI) are one of the biological hazards associated with injection use. In a study conducted in a tertiary care hospital of Pakistan, it was witnessed that most of the NSIs (more than 47%) were from disposable needles and most commonly (33%) occurred during recapping of needle [6]. Similarly, a study from Pokhara, Nepal revealed that recapping needle (25%) was the important cause of NSI and 56% of NSI were reported. Surprisingly, 80% of respondents thought needle recapping was necessary and should be carried out [13]. Discouraging needle recapping and proper disposal of sharps and needles is a very important preventive step for NSI [22]. Our study found that two handed recapping of needle was not practised in the health centre. One of the HCWs reported that he does one hand recapping as and when required. They avoided recapping because it was described as hazardous in various trainings related to injectable vaccine administration. Only a few of them knew correct reason for avoiding recapping. Two in-charges reported an average of 1.5 needle stick injuries (NSI) per healthcare worker during the past six months.

Improper disposal of syringes and incidences of NSIs show that there is a wide gap between provider knowledge and their practice hence injection related behavioural changes are required which can be brought by proper training and regular supervision. In the Syrian Arab Republic, after training the HCWs in injection safety and waste management their behaviour changed, needle recapping significantly reduced and safety boxes use increased [19].

Among the diseases transmitted by NSI, hepatitis B is most contagious but fortunately a vaccine is available [5,6]. Hepatitis B vaccination to HCWs is very important in countries like Nepal, where hepatitis B is a major public health concern with 200 thousand carriers and the disease accounts for 6% of acute hepatitis [23]. In our study all the participants reported that they had received full course (three doses at 0, 1 & 6 months) of hepatitis B vaccine which is better than the condition reported in a study done in Pokhara city [13] where only 71% of HCWs (including doctors) received vaccination. The vaccination in Baglung was done few years back by a non-governmental organization (NGO) for which the HCWs were not charged. Now they require a booster dose of the vaccine to maintain the immunity but a NGO with such programme is not present so government perusal in the issue is required.

The main waste disposal technique for disposing used injection equipment was incineration (burning) in a pit (80%) Figure 2. The practices of uncontrolled open burning and burial in a pit were observed in two health facilities. Fifty per cent of in-charges shared that there are designated staffs for bio-medical wastes handling and disposal in their health facility but none of these staff had received any formal training in waste management. The designated staffs dispose the bio-medical waste without wearing any protective materials such as gloves, mask, boot, apron etc and without any safety measures. The office staff (attender) who dispose the biomedical wastes often accept the biohazards exposure as a part of their job, which might be due to lack of awareness about the associated dangers. Moreover, BBVs transmitted through unsafe injection practice (including NSI) remain silent (grow slowly) for many years so the dangerous effects are not observed soon. Hence the occupational safety hazard might not be taken seriously [24]. Contrary to the lack of health safety training and preventive measures for health waste disposal, none of the staff reported to have suffered injury from sharp wastes.

Disposal of single use injection equipment is a major problem. Most of the staff who handle the waste are not trained and the staff who are aware of the hazards are negligent [25]. Hence, urgent requirement of allocation of trained staff and mechanism for management, supervision and enforcement of disposal procedures is required. Same type of recommendation was made by Bhattarai et al a decade back [25]. During our study, we observed that incinerator was also being constructed in new governmental health facilities under construction. It would be better if all health facilities could be equipped with an incinerator so disposal item may be burnt quickly and easily every day.

### Knowledge with regard to safe injection practice

Health care workers were aware of the risk of infection with blood borne pathogens associated with NSI. All were aware about at least one pathogen transmitted through unsafe injection practices and NSI (Table 1). We found the HCWs were aware of the risk associated with NSI and have some knowledge of pathogens transmitted by NSI. Even though it might be Hawthorne effect, we found that the HCWs, during our study and interactions, were eager to learn proper injection safety measures and adopt them to make their practice safer. Respondent may respond/act differently, if they feel they are being watched or study is being done on their activity or response. This changed response is called Hawthorne effect [26]. Due to geographic isolation, the opportunities for HCWs to engage in professional development and monitoring of their practice were limited. Hence, safe injection practice related changes were slow to be incorporated.

**Table 1:** Disease that may be transmitted through unsafe injection practice according to HCWs studied

**Disease transmitted**	**n (%)**
HIV	9 (90%)
Hepatitis (including Hepatitis B)	8 (80%)
Syphilis	2 (20%)
Typhoid fever	1 (10%)
Tuberculosis	1 (10%)

Most injection providers (90%) reported that none of them had been sent for training on safe injection practice in the last two years. A training solely dedicated to injection safety is needed to bring about positive changes in their attitude regarding safe injection practice. A health safety wing within department of health services, Ministry of Health and a regular curriculum in health safety and health waste disposal for the primary health care worker is needed.

Washing hands with soap and administration of Tetanus Toxoid vaccine was the only safety measure adopted after NSI. None of the health facility has guideline or standard operating procedure for post NSI. Eighty per cent reported that the health facilities had a written injection safety management policy but no one could show it to the investigators. Lack of guidelines for safety practice and medical waste management may result in unsafe injection practice so it has to be addressed.

### Limitations

Even though the current study provides important baseline information to aid in the formulation of relevant policies and future research, it has limitations as well. The study cannot be generalized to Baglung district because district level hospital, private health facilities and informal health care providers e.g. medical shopkeeper, traditional healers etc (who do not have legal right and training to administer injections but provide it in practice) were not included in the study. The study would have been more representative if we could have included more number of health facilities. The research was a preliminary study and there was geographical hindrance along with other constraints, so the sample size could not be increased. The study would have given a clearer picture if audit of prescriptions were also done. It could not be done due to lack of time and improper documentation.

## Conclusion

The study highlights the need for a detailed study on safe injection practice and HCWs safety. It was found that the government of Nepal is directly and indirectly endeavouring to promote safe injection practices by implementing the widespread use of single use disposable injection equipment, supplying safety box and providing training on rational use of few injections. But monitoring and supervision is poor and there remain a number of grey areas where unsafe injection practices persists placing HCWs and the community at risk of associated hazards.

## Abbreviations

AHW: Auxiliary health worker; AIDS: Acute Immunodeficiency Syndrome; ANM: Auxiliary nursing midwife; HA: Health assistant; HBV: Hepatitis B virus; HCV: Hepatitis C virus; HCWs: Health care workers; HIV: Human Immunodeficiency Virus; HP: Health post; NGO: Non-governmental organization; NSI: Needle Stick Injury; PHC: Primary Health Centre; SD: Standard deviation; SHP: Sub health post; VDCs: Village development committees; WHO: World Health Organization.

## Competing interest

The authors declare that they have no competing interests.

## Authors' contributions

SG and DSR conceived and designed study. SG, DSR and PRS finalized the methodology and tools used. SG and BKC collected data, analysed and drafted the manuscript. All the authors made significant contributions in manuscript writing and finalizing the manuscript. The final manuscript have been read and approved by all the authors.

## Pre-publication history

The pre-publication history for this paper can be accessed here:

http://www.biomedcentral.com/1472-698X/13/3/prepub
